# Effects of Quadriceps Strength Recovery in the First Year After Anterior Cruciate Ligament Reconstruction on Gait Biomechanics

**DOI:** 10.1177/19417381261479680

**Published:** 2026-09-11

**Authors:** Sergio A. Lemus, Christin Büttner, Liubov Arbeeva, Caroline Lisee, Natália Favoreto, R. Alexander Creighton, Ganesh M. Kamath, Jeffrey T. Spang, Christopher D. Ingersoll, Samantha Tayne, Joe M. Hart, J. Troy Blackburn, Brian Pietrosimone

**Affiliations:** Department of Exercise and Sport Science, University of North Carolina at Chapel Hill, Chapel Hill, North Carolina; Department of Exercise and Sport Science, University of North Carolina at Chapel Hill, Chapel Hill, North Carolina, and Institute of Human Movement Science and Health, Chemnitz University of Technology, Chemnitz, Germany; Thurston Arthritis Research Center, University of North Carolina at Chapel Hill, Chapel Hill, North Carolina; Department of Kinesiology, University of Georgia, Athens, Georgia; Department of Exercise and Sport Science, University of North Carolina at Chapel Hill, Chapel Hill, North Carolina; Department of Orthopaedics, University of North Carolina at Chapel Hill, Chapel Hill, North Carolina; Department of Orthopaedics, University of North Carolina at Chapel Hill, Chapel Hill, North Carolina; Department of Orthopaedics, University of North Carolina at Chapel Hill, Chapel Hill, North Carolina; Department of Health Sciences, University of North Carolina at Chapel Hill, Chapel Hill, North Carolina; Department of Orthopaedics, University of North Carolina at Chapel Hill, Chapel Hill, North Carolina; Department of Orthopaedics, University of North Carolina at Chapel Hill, Chapel Hill, North Carolina; Department of Exercise and Sport Science, University of North Carolina at Chapel Hill, Chapel Hill, North Carolina; Department of Exercise and Sport Science, University of North Carolina at Chapel Hill, Chapel Hill, North Carolina

**Keywords:** knee, post-traumatic osteoarthritis, rehabilitation, quadriceps strength, walking biomechanics

## Abstract

**Background:**

Aberrant walking biomechanics, a factor linked to knee osteoarthritis (KOA), are common after anterior cruciate ligament (ACL) injury. Although quadriceps weakness often persists after ACL reconstruction (ACLR), the extent to which strength recovery influences gait biomechanics over the first postoperative year, a critical window for implementing rehabilitation strategies, remains unclear.

**Hypothesis:**

Distinct quadriceps strength recovery trajectories will emerge after ACLR, with the lesser strength recovery group exhibiting more aberrant gait biomechanics compared with the greater strength recovery group and controls.

**Study Design:**

Prospective cohort study.

**Level of Evidence:**

Level 2.

**Methods:**

Of 62 enrolled patients with a primary ACL injury, 59 were eligible and matched with uninjured controls by sex, age, and body mass index (BMI). Quadriceps strength was assessed preoperatively and at 4, 6, and 12 months post-ACLR, and gait biomechanics, including vertical ground reaction force (vGRF), knee flexion angle (KFA), knee extension moment (KEM), and knee adduction moment (KAM), were assessed at 4, 6, and 12 months post-ACLR. Group-based trajectory modeling identified strength recovery profiles, and functional mixed effects modeling compared gait biomechanics between strength trajectory groups and controls.

**Results:**

Two distinct strength trajectories were identified. Both the weaker (N = 31) and stronger (N = 28) ACLR groups demonstrated lesser vGRF during early stance compared with controls, with maximum differences reaching −0.15 body weight (BW). Compared with the stronger group, the weaker group exhibited lesser KFA (–1.85° and −2.18° at 6 and 12 months) and KEM (0.006 BW × height at 4 and 6 months) during early stance. Compared with healthy controls, both ACLR groups exhibited lesser KFA and KEM during early stance across postoperative timepoints, with larger and more persistent differences observed in the weaker group. Only the weaker group had significantly lower KAM relative to controls (0.006 to 0.007 BW × height at 4, 6, and 12 months).

**Conclusion:**

Although the stronger group exhibited more dynamic gait waveforms than the weaker group, walking biomechanics were not fully normalized to those of healthy controls.

**Clinical Relevance:**

These findings highlight the need for complementary interventions to strength training during the first year post-ACLR to target aberrant biomechanics and potentially reduce long-term KOA risk.

Aberrant walking biomechanics commonly develop after anterior cruciate ligament (ACL) injury,^9,12^ with patients showing bilateral differences in key biomechanical gait outcomes relative to uninjured controls. These differences are observed at multiple timepoints from the preoperative period through 12 months post-ACL reconstruction (ACLR),^9^ and in some cases persist up to 2 years.^32^ Specifically, after ACLR, patients commonly exhibit lesser peak vertical ground reaction force (vGRF), greater vGRF during midstance, and lesser peak knee adduction moment (KAM) during the stance phase of gait compared with uninjured controls, reflecting a less dynamic loading pattern. This pattern has been associated with early tibiofemoral cartilage matrix degeneration detected using T1ρ magnetic resonance imaging in the first 6 to 12 months after ACLR, underscoring the pathological relevance of specific biomechanical phenotypes.^6,16^ Notably, vGRF loading profiles observed in the first 6 months post-ACLR resemble those exhibited by older adults with radiographically confirmed knee osteoarthritis (KOA),^4^ supporting concerns that post-ACLR gait may accelerate degenerative joint changes. Importantly, early structural and biochemical joint tissue alterations may already be present after ACL injury and ACLR, and may influence gait biomechanics before KOA develops.^17,25^ Together, these findings underscore the importance of identifying factors that drive these aberrant biomechanical profiles early in recovery. One such factor may be quadriceps weakness, as it has been associated with more pronounced aberrations in walking biomechanics when comparing people with lower versus higher quadriceps strength post-ACLR,^26^ potentially increasing the risk of long-term KOA development.

Quadriceps weakness is one of the most common clinical impairments after ACLR,^33^ and is a primary target in rehabilitation efforts.^24^ Deficits in quadriceps strength, often measured as knee extension torque of the ACLR limb normalized to body mass (Nm·kg^–1^), are consistently linked to poor patient-reported outcomes^27^ and may serve as a predictive marker for future KOA development.^24,29^ Mechanistically, quadriceps dysfunction has been associated with reduced dynamic knee control during the weight acceptance phase of the stance (early to midstance), which may contribute to less knee flexion and reduced sagittal plane loading during this phase.^2,20^ In addition, people with lesser quadriceps strength exhibit lower peak vGRF during early stance at 1 year postoperatively.^2,7^ While improvements in quadriceps strength are often achieved through formal rehabilitation, many patients post-ACLR continue to exhibit abnormal gait patterns, suggesting that strength restoration alone may not be sufficient to fully restore normal walking biomechanics.^7,20^

Clinical benchmarks for quadriceps strength recovery after ACLR often reference an absolute isometric strength value of ≥3.0 Nm·kg^–1 19,26,27^ and a limb symmetry index (LSI) >90%.^36^ Accordingly, it remains unclear to what extent recovery of quadriceps strength, whether defined by attainment of an absolute strength target or symmetry criterion, translates to normalization of gait biomechanics and thus lower KOA risk. This distinction is important, as the persistence of abnormal gait patterns after strength restoration suggests the involvement of additional causal factors, such as motor learning deficits, highlighting the need for targeted gait retraining as a complementary focus in rehabilitation.^14,22^ While cross-sectional studies have shed light on the associations between quadriceps strength groups and aberrant gait biomechanics,^2,26^ they fail to capture the longitudinal variability of gait adaptations throughout the stance phase from the preoperative period through 12 months post-ACLR, which represents a critical window for identifying gait abnormalities that may persist after discharge from formal rehabilitation and contribute to posttraumatic KOA risk. To effectively design and implement interventions aimed at correcting these aberrant gait biomechanics, it is first necessary to understand how quadriceps strength recovery over time influences gait biomechanics throughout the first year post-ACLR.

Therefore, the purpose of this study was to: (1) identify quadriceps strength recovery trajectory profiles in the ACLR limb, while accounting for contralateral limb strength, using longitudinal data collected from the preoperative period through 12 months post-ACLR; and (2) determine whether gait biomechanical profiles, including vGRF, knee flexion angle (KFA), knee extension moment (KEM), and KAM, at 4, 6, and 12 months post-ACLR differed between strength trajectory groups and between each strength trajectory group and matched uninjured controls. Given the differences between ACLR patients and uninjured controls commonly observed around the first vGRF peak and during midstance^4,30^ these portions of stance were of particular interest when interpreting waveform patterns between groups. Accordingly, we hypothesized that distinct strength trajectory profiles would emerge, with patients exhibiting lesser strength recovery showing more aberrant gait biomechanics, characterized by less dynamic vGRF, KFA, KEM, and KAM waveforms, compared with those exhibiting greater strength recovery and uninjured controls.

## Methods

### Study Design

We conducted a prospective, longitudinal cohort study with a nested case-control design to evaluate bilateral quadriceps strength in people with primary unilateral ACL injury at 4 timepoints: preoperatively, and at 4, 6, and 12 months after ACLR. Strength data were used to stratify participants into quadriceps strength trajectory groups. Gait biomechanics in the ACLR limb, including vGRF, KFA, KEM, and KAM, were assessed at habitual walking speed at 4, 6, and 12 months post-ACLR and compared with matched uninjured control data collected at a single timepoint.

### Participants

We enrolled 62 participants (27 men, 35 women; age, 21.8 ± 4.6 years; body mass index [BMI], 24.1 ± 3.2 kg·m^–2^) with a primary unilateral ACL injury. Eligible participants (N = 59) were 16 to 35 years old and underwent primary unilateral arthroscopic ACLR using a bone-patellar tendon-bone, quadriceps, or hamstring autograft. Participants were excluded if they received an allograft, required revision ACLR or multiligament surgery, sustained a lower extremity fracture during their ACL injury, had a previous diagnosis of KOA or other knee joint disease, were pregnant at the time of enrollment, or intended to become pregnant during the study period. All ACLR participants engaged in supervised rehabilitation for a minimum of 6 months postsurgery.

Uninjured controls were enrolled to match each ACLR participant. Control participants were eligible if they had no history of lower extremity orthopaedic surgery or knee injury, no joint injury or concussion within the past 6 months, no diagnosis of inflammatory arthritis, and no condition that could affect gait or physical activity. Controls were matched to ACLR participants based on sex (male/female), baseline age (±2 years), and BMI (±4 kg·m^–2^). For all participants, demographic data (e.g., height, weight, age) were recorded at each testing session. Study procedures were approved by the Institutional Review Board at the University of North Carolina at Chapel Hill (Study no. 20-2227), and all participants provided written informed consent or assent with guardian permission when appropriate.

### Strength Measurements

Quadriceps strength was assessed in both the ACLR and contralateral limbs via an isokinetic dynamometer (HUMAC NORM; CSMi) following protocols described in previous studies.^15,21^ These protocols have demonstrated strong intratester (intraclass correlation coefficient [ICC] = 0.98) and intertester (ICC = 0.97) reliability for maximal voluntary isometric contraction (MVIC) measurement.^21^ MVICs were used to obtain peak torque, which was normalized to body mass (Nm·kg^–1^). Participants were seated with the hip flexed to 85° and the knee to 90°, and the lower limb was secured to the dynamometer arm using a padded Velcro strap placed at the gastrocnemius-soleus junction.

After a graded warm-up consisting of submaximal efforts at 25%, 50%, and 75% of perceived maximum, with 60 seconds of rest provided between each effort, participants performed practice MVIC trials until torque values between 2 consecutive trials stabilized within a 5% margin. The average of the final 2 practice MVICs was used to generate a visual feedback target line, set at 10% above this average, to motivate maximal exertion. Participants viewed real-time torque output during testing and were instructed to exceed the visual target line. Standardized verbal encouragement was provided during all trials. Two valid MVICs were recorded per limb. A trial was considered valid if torque output met or exceeded the average from the practice trials. Rest intervals of ≥60 seconds were provided between MVIC attempts to minimize fatigue. The contralateral limb was always tested first, followed by the ACLR limb. The highest peak torque value from the 2 valid trials per limb was used for analysis to reflect each participant’s true maximal voluntary strength capacity and to maintain consistency with the standardized strength assessment protocol used in previous work.^15,21^

### Biomechanical Gait Assessment

Gait biomechanics were collected using a motion capture system (Vicon Nexus Version 2.15; Vicon Motion Systems Ltd) and a standardized overground walking protocol described in previous studies.^8-10^ Participants walked barefoot at their self-selected habitual walking speed along a 6-meter walkway. During each trial, 10 high-speed infrared cameras (Vicon Bonita; Vicon Motion Systems Ltd) captured the trajectories of 29 reflective markers placed on anatomical landmarks of the lower limbs, pelvis, and trunk. Simultaneously, ground reaction forces were recorded using 3 embedded force plates (FP406010; Bertec Corp). Habitual gait speed was determined from a minimum of 5 test trials using 2 timing gates (Dashr) positioned 0.97 meters apart. After this, 5 successful gait trials were collected from both limbs. A trial was considered successful if the participant walked within ±5% of their average habitual speed and achieved full-foot contact on the force plate during stance (i.e., from heel strike to toe-off). Data were processed using custom scripts in Visual3D (Version 2020; C-Motion) and MATLAB (Version R2022b; MathWorks). Marker and force plate signals were filtered using a recursive fourth-order low-pass Butterworth filter with a cut-off frequency of 10 Hz. Walking mechanics outcomes, including vGRF, KFA, KEM, and KAM, were time-normalized to 101 data points, representing 0% to 100% of the stance phase, defined from heel strike (vGRF, >20 N) to toe-off (vGRF, <20 N). Net internal knee moments were calculated using inverse dynamics.

### Statistical Analysis

#### Strength Trajectory Modeling

Quadriceps strength data were available for ≥1 timepoint for 59 eligible participants who underwent ACLR surgery (Table 1). To identify subgroups with distinct strength recovery patterns in the ACLR limb, we applied group-based trajectory modeling using PROC TRAJ in SAS (SAS Institute Inc). This approach fits a semiparametric (discrete) mixture model to longitudinal data using maximum likelihood method. Each participant was assigned to a latent trajectory group based on the highest posterior probability of membership. The optimal number of groups was determined using the Bayesian Information Criterion (BIC) and group size (>10% of sample). The shapes of trajectories were determined based on the significance of polynomial terms. To account for contralateral limb strength, the corresponding value from the uninvolved limb at each timepoint was included as a time-varying covariate in the final model. This approach allows recovery of the involved limb to be evaluated while accounting for bilateral strength changes post-ACLR. Consistent with model assumptions of conditional independence and an appropriate outcome distribution, the identified trajectories are interpreted as heterogeneous recovery patterns rather than fixed subgroups.

**Table 1.** Participant availability and reasons for missing data across timepoints

Timepoint	Strength data, n	Gait data, n	Notes about missing participants
Enrollment (consented)	-	-	62 consented; 2 withdrew after enrollment; 1 did not choose ACLR
Preoperative	49	-	8 unable to perform assessment; 1 technical issue; 1 missed appointment
ACLR surgery (final surgical cohort)	59	59	59 underwent ACLR; 1 received an allograft and was excluded
4 months post-ACLR	55	55	3 missed appointments
6 months post-ACLR	53	53	1 technical issue; 3 missed appointments; 1 lost to follow-up
12 months post-ACLR	50	50	2 technical issues; 2 missed appointments; 1 ineligible (pregnancy); 1 lost to follow-up

Enrollment reflects consent and screening only (no strength or gait testing was performed at that time). Strength data were collected preoperatively and at 4, 6, and 12 months after ACLR. Gait biomechanics were assessed postoperatively only. ACLR, anterior cruciate ligament reconstruction.

Group-level descriptive statistics, reported as means and standard deviations for continuous variables and as N (%) for categorical variables, were calculated for sex, age, BMI, and gait speed across the ACLR strength trajectory groups and the uninjured control group. Additional variables, including days before ACLR (for preoperative assessment), days since ACLR (for postoperative timepoints), graft type, presence of meniscal injury, and Tegner activity scores, were summarized for the ACLR strength groups only. Continuous variables were analyzed using a 1-way analysis of variance (ANOVA). Group differences in age and BMI were assessed at the preoperative timepoint, gait speed comparisons were conducted across all postoperative timepoints, whereas Tegner scores were compared at only the 6- and 12-month post-ACLR timepoints. If normality assumptions were violated, the Kruskal-Wallis test was performed. When a significant main effect was detected, Tukey’s post hoc test was used for pairwise comparisons. Differences in categorical variables, including the occurrence of medial meniscal injury, lateral meniscal injury, and chondral lesions, were assessed using chi-square tests. Interlimb symmetry was quantified using an LSI, calculated as the ratio of involved to uninvolved quadriceps strength multiplied by 100. Symmetry was compared between the stronger and weaker ACLR strength trajectory groups at each timepoint (preoperative, 4, 6, and 12 months post-ACLR) using Welch’s independent-samples *t* tests. Descriptive statistics and non-waveform inferential analyses were performed using Python (Version 3.11; Python Software Foundation).

#### Gait Biomechanical Analysis

Gait biomechanical data were available for ≥1 timepoint for 59 eligible ACLR participants (Table 1). Functional waveform analysis was used to assess stance-phase differences in vGRF, KFA, KEM, and KAM between quadriceps strength trajectory groups and controls, using methods consistent with previous studies.^8,10^ At each timepoint, separate functional mixed effects models were constructed to evaluate differences in each biomechanical outcome. Average waveforms from 5 successful walking trials per participant were modeled using Bayesian functional regression, with B-spline basis functions to derive representative waveforms for each group. Each model estimated the mean difference between group waveforms along with corresponding 95% CIs and Cohen’s *d* effect sizes. Effect size thresholds were interpreted as follows: weak (Cohen’s *d* ≈ 0.2), moderate (Cohen’s *d* ≈ 0.5), and strong (Cohen’s *d* ≈ 0.8). Time regions in which the 95% CI of the group difference did not cross zero were considered statistically significant. All waveform analyses were performed using the FunctionalMixedEffects package in R (Version 4.1.2, R Foundation for Statistical Computing) through RStudio.

The primary analyses were conducted without controlling for gait speed to characterize how ACLR participants walked under self-selected conditions, as slower walking speed may represent part of the clinical presentation rather than only a confounding factor. However, because habitual gait speed differed between ACLR and control participants at all timepoints, and given the established association between walking speed, gait biomechanics, and post-ACLR KOA risk,^11,35^ an additional set of analyses was conducted controlling for individual gait speed at each timepoint, normalized to the sample mean at that timepoint. These gait speed-adjusted models followed the same statistical framework and were conducted for all primary biomechanical outcomes at each postoperative timepoint.

## Results

### Strength Recovery Trajectories

**Figure 1.**
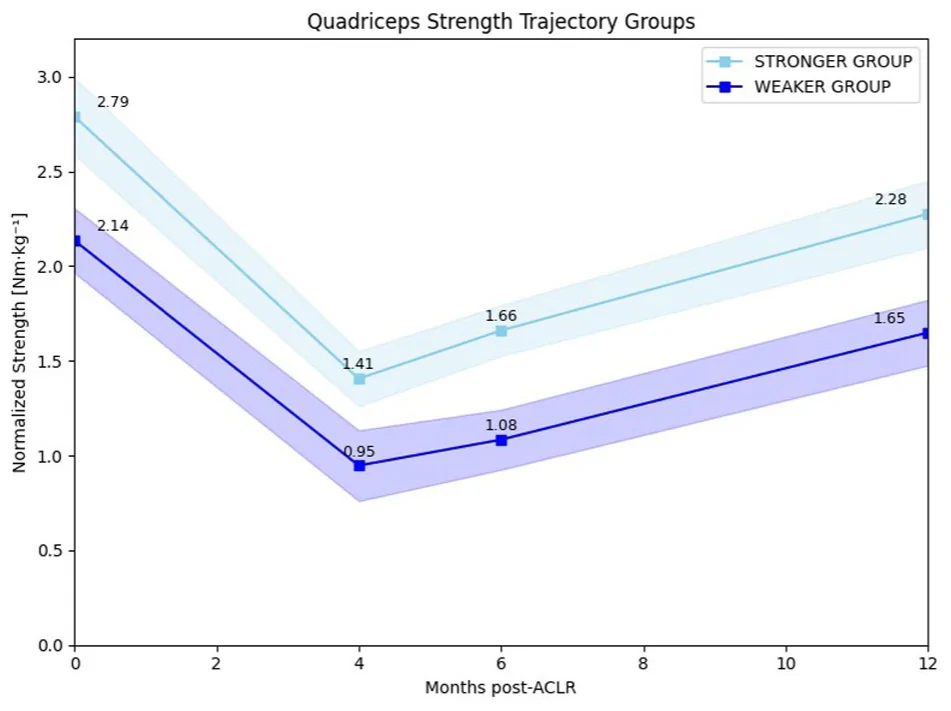
Quadriceps strength recovery trajectories for the involved limb from preoperative assessment through 12 months post-ACLR. Two distinct strength trajectory groups were identified using group-based trajectory modeling: a stronger group (light blue) and a weaker group (dark blue). Lines represent predicted normalized quadriceps strength (Nm·kg^–1^) across timepoints, and shaded regions indicate 95% confidence intervals. Strength values were derived from MVIC testing and normalized to body mass. ACLR, anterior cruciate ligament reconstruction; MVIC, maximal voluntary isometric contraction.

The final model identified 2 distinct quadratic trajectories for muscle strength in the involved limb (Figure 1). Alternative trajectory solutions were evaluated, but a 3-group model yielded only 3 participants (5.2%) in the smallest group; therefore, the 2-group solution was retained. Participants in the trajectory group with higher strength values were classified as the stronger group (N = 28), while those in the lower trajectory were classified as the weaker group (N = 31).

### Anthropometrics, Gait Speed, and ACLR-Related Outcomes

No statistically significant differences were observed in age or BMI among the weaker ACLR group, stronger ACLR group, and controls. However, both ACLR strength trajectory groups walked slower than controls (1.36 ± 0.14 m·s^–1^) at 4 (weaker, 1.21 ± 0.16 m·s^–1^; stronger, 1.26 ± 0.10 m·s^–1^), 6 (weaker, 1.22 ± 0.14 m·s^–1^; stronger, 1.28 ± 0.10 m·s^–1^), and 12 months (weaker, 1.26 ± 0.11 m·s^–1^; stronger, 1.28 ± 0.11 m·s^–1^) post-ACLR (Table 2). No statistically significant differences were found between ACLR strength groups in Tegner activity levels or the occurrence of meniscal injuries or chondral lesions. Interlimb quadriceps strength symmetry differed significantly between ACLR strength trajectory groups at all assessed timepoints (Table 2), with the stronger group demonstrating 20% to 25% higher LSI values than the weaker group across time. At 12 months post-ACLR, the stronger group exhibited a mean LSI of 91.1 ± 11.7%, whereas the weaker group exhibited 66.8 ± 16.9%. However, only 3 ACLR participants (5.1%) achieved an involved-limb quadriceps strength threshold of 3.0 Nm·kg^–1^ at 12 months post-ACLR.

**Table 2.** Anthropometrics, gait speed, and ACLR-related characteristics across quadriceps strength and control groups

Characteristics	Timepoint	Weaker group	Stronger group	Controls	*P* value
Sex, n (male/female)		11/20	14/14	26/33	
Age, years		22.4 ± 4.8	21.3 ± 4.6	19.4 ± 3.5	0.45
BMI, kg·m^–2^		24.5 ± 3.5	23.9 ± 2.9	22.1 ± 3.4	0.44
Gait speed, m·s^–1^	4 months	1.21 ± 0.16*^b^*	1.26 ± 0.10*^b^*	1.36 ± 0.14*^a^*	<0.001*
	6 months	1.22 ± 0.14*^b^*	1.28 ± 0.10*^b^*	1.36 ± 0.14*^a^*	<0.001*
	12 months	1.26 ± 0.11*^b^*	1.28 ± 0.11*^b^*	1.36 ± 0.14*^a^*	0.001*
Days before ACLR	Preop	9.3 ± 8.4	11.2 ± 10.5		
Days since ACLR	4 months	110.8 ± 23.4	101.9 ± 23.4		
	6 months	173.5 ± 8.4	174.4 ± 12.2		
	12 months	352.9 ± 21.0	331.4 ± 72.2		
Graft type, n (PT/QT/H)		29/0/2	26/1/1		
Medial meniscal injury, %		29.0	17.9		0.48
Lateral meniscal injury, %		77.4	57.1		0.17
Chondral injury, %		32.3	25.0		0.74
Tegner activity level scale	6 months	5.3 ± 1.8	6.0 ± 2.1		0.15
	12 months	7.1 ± 2.1	7.8 ± 2.3		0.34
Interlimb symmetry (LSI), %	Preop	65.0 ± 14.2	86.3 ± 13.9		<0.001*
	4 months	49.5 ± 26.9	68.0 ± 14.1		0.003*
	6 months	54.7 ± 28.9	78.1 ± 13.7		0.001*
	12 months	66.8 ± 16.9	91.1 ± 11.7		<0.001*

Data are reported as group mean ± SD, unless otherwise stated. ANOVA, Kruskal-Wallis test, chi-square test, and Welch’s independent-samples *t* test *P* values are presented as appropriate. For rows where only ACLR strength groups are included (e.g., Tegner scores, surgical characteristics, and interlimb symmetry), the *P* value reflects comparisons between weaker and stronger groups only. “Stronger” and “weaker” labels reflect relative quadriceps strength trajectory profiles within the ACLR cohort and do not indicate recovery to normative or clinically sufficient strength levels. ACLR, anterior cruciate ligament reconstruction; ANOVA, analysis of variance; BMI, body mass index; H, hamstring; LSI, limb symmetry index; Preop, preoperatively; PT, patellar tendon; QT, quadriceps tendon.

a,b Different superscripts indicate statistically significant differences between groups. *Statistically significant effects (*P* < 0.05).

### Gait Biomechanical Outputs

#### Vertical Ground Reaction Force

**Figure 2.**
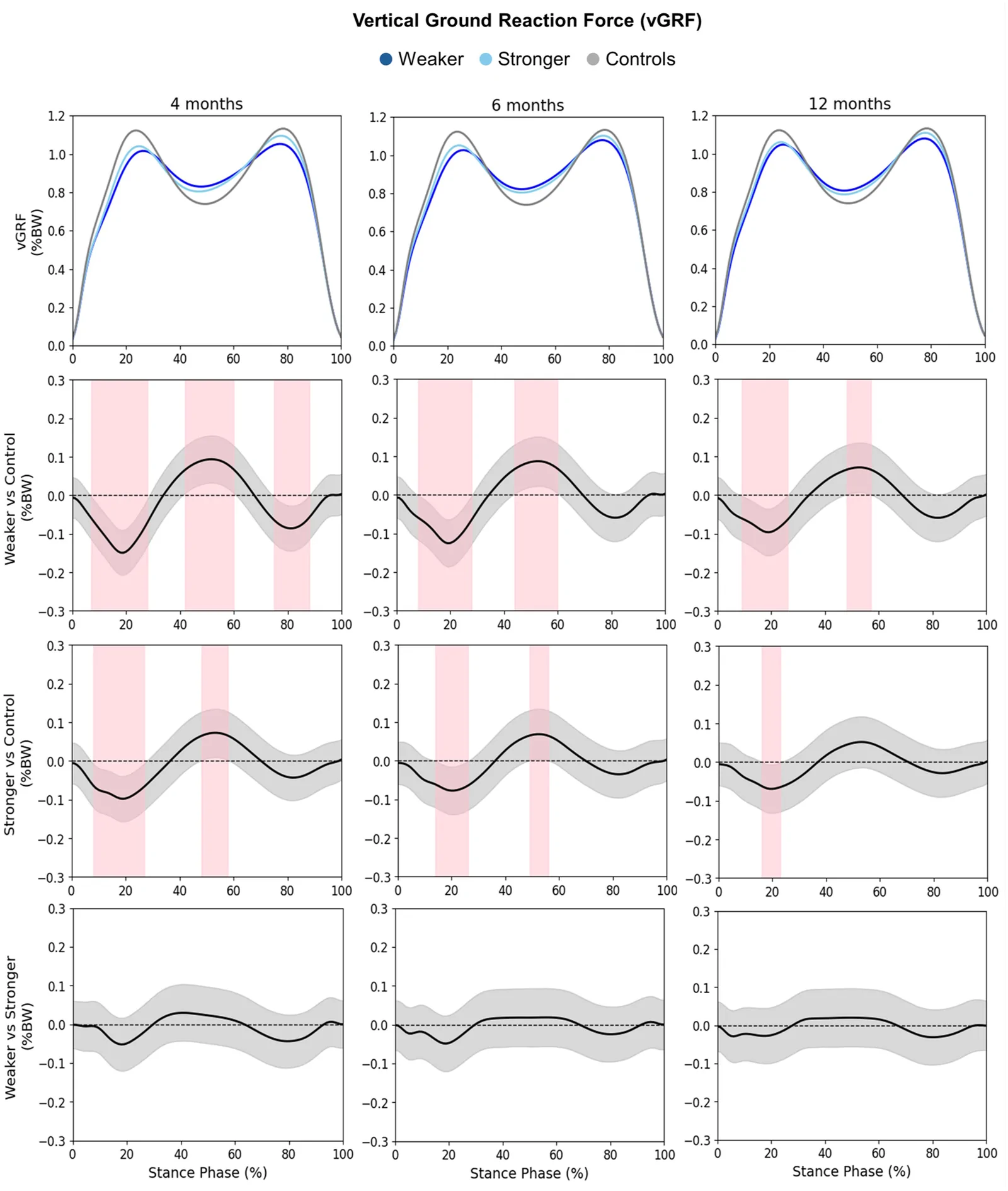
Average vGRF waveforms and corresponding mean differences with 95% confidence intervals (shaded in gray) across gait stance phase for the weaker ACLR group, stronger ACLR group, and uninjured control group at the 4-, 6-, and 12-month timepoints after ACLR. The first row displays mean vGRF waveforms for each group; the second row shows differences between the weaker ACLR group and controls; the third row compares the stronger ACLR group with controls; and the fourth row compares the weaker and stronger ACLR groups. The first, second, and third columns correspond to the 4-, 6-, and 12-month assessments, respectively. Statistically significant group differences, defined as intervals where the 95% CIs of the mean differences do not include zero, are highlighted in pink. ACLR, anterior cruciate ligament reconstruction; BW, body weight; vGRF, vertical ground reaction force.

No statistically significant differences were found between the stronger and weaker ACLR groups at any phase of stance across all timepoints (Figure 2 and Table 3). Both groups, however, showed lesser vGRF during early stance compared with healthy controls at 4, 6, and 12 months post-ACLR, followed by greater vGRF during midstance at several timepoints. Notably, only the weaker group exhibited lesser vGRF during 75% to 88% of stance, corresponding to the second peak vGRF, at 4 months post-ACLR compared with healthy controls.

**Table 3.** Results of waveform analyses of vGRF at 4-, 6-, and 12-month timepoints after ACLR

Comparison	Timepoint	Areas of difference, %	Maximum difference, (BW)	Cohen’s *d* effect size
(1) Weaker – controls	4 months	7-28	–0.15	–1.54
		42-60	0.09	1.28
		75-88	–0.09	–1.40
	6 months	8-28	–0.13	–1.29
		44-60	0.09	1.19
	12 months	9-26	–0.10	–1.01
		48-57	0.07	0.98
(2) Stronger – controls	4 months	8-27	–0.10	–1.04
		48-58	0.07	0.99
	6 months	14-26	–0.08	–0.83
		49-56	0.07	0.93
	12 months	16-23	–0.07	–0.73
(3) Weaker – stronger	4 months	-	-	-
	6 months	-	-	-
	12 months	-	-	-

Comparisons were made between (1) the weaker ACLR group and healthy controls, (2) the stronger ACLR group and healthy controls, and (3) the weaker and stronger ACLR groups, including the gait stance regions with statistically significant differences, the peak differences observed in those regions, and the corresponding average Cohen’s *d* effect sizes for the significant intervals. For effect size thresholds, see Methods. “Stronger” and “weaker” labels reflect relative quadriceps strength trajectory profiles within the ACLR cohort and do not indicate recovery to normative or clinically sufficient strength levels. Dashes indicate no statistically significant between-group differences for that comparison at the given timepoint (*P* ≥ 0.05). ACLR, anterior cruciate ligament reconstruction; BW, body weight; vGRF, vertical ground reaction force.

#### Knee Flexion Angle

**Figure 3.**
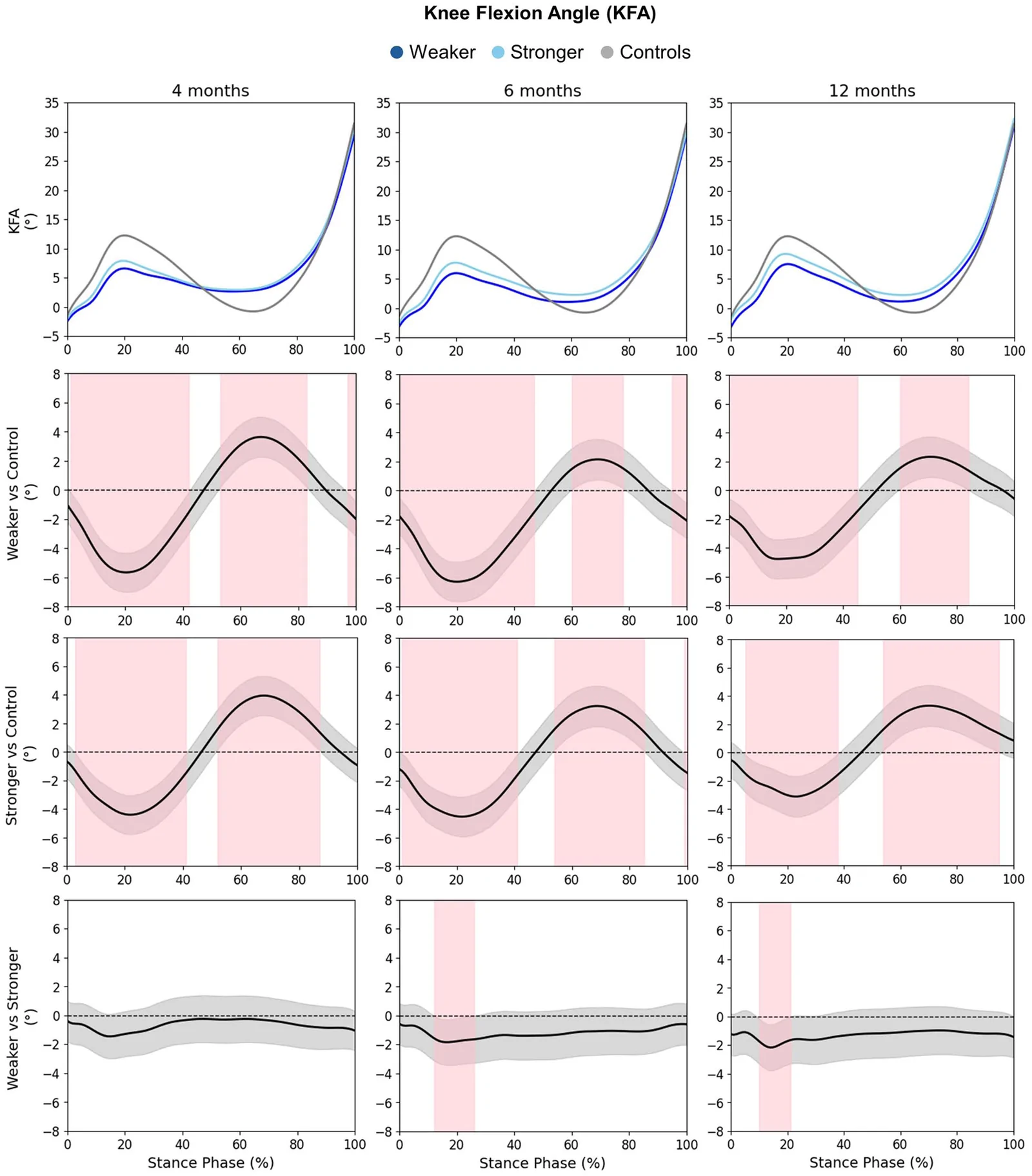
Average KFA waveforms and corresponding mean differences with 95% confidence intervals (shaded in gray) are presented across the gait stance phase for the weaker ACLR group, stronger ACLR group, and uninjured control group at the 4-, 6-, and 12-month timepoints after ACLR. The first row displays mean KFA waveforms for each group; the second row shows differences between the weaker ACLR group and controls; the third row compares the stronger ACLR group with controls; and the fourth row compares the weaker and stronger ACLR groups. The first, second, and third columns correspond to the 4-, 6-, and 12-month assessments, respectively. Statistically significant group differences, defined as intervals where the 95% CIs of the mean differences do not include zero, are highlighted in pink. ACLR, anterior cruciate ligament reconstruction; KFA, knee flexion angle.

The stronger group showed greater KFA during early stance at 6 (12% to 26%) and 12 (10% to 21%) months compared with the weaker ACLR group (Figure 3 and Table 4). Both ACLR groups exhibited reduced KFA during the first half of stance relative to healthy controls across all post-ACLR timepoints, followed by greater KFA during mid- to late-stance. Statistically significant differences during late stance were also observed in both ACLR groups compared with controls.

**Table 4.** Results from waveform analyses of KFA 4-, 6-, and 12-month timepoints after ACLR

Comparison	Timepoint	Areas of difference, %	Maximum difference, deg	Cohen’s *d* effect size
(1) Weaker – controls	4 months	1-42	–5.77	–0.96
		53-83	3.64	0.77
		97-100	–2.00	–0.39
	6 months	0-47	–4.92	–1.12
		60-78	3.54	0.48
		95-100	–2.06	–0.38
	12 months	0-45	–4.77	–0.92
		60-84	2.33	0.52
(2) Stronger – controls	4 months	3-41	–4.40	–0.79
		52-87	3.94	0.87
	6 months	1-41	–4.53	–0.80
		54-85	3.24	0.73
		99-100	–1.46	–0.32
	12 months	5-38	–3.12	–0.53
		54-95	3.32	0.69
(3) Weaker – stronger	4 months	-	-	-
	6 months	12-26	–1.85	–0.34
	12 months	10-21	–2.18	–0.45

Comparisons were made between (1) the weaker ACLR group and healthy controls, (2) the stronger ACLR group and healthy controls, and (3) the weaker and stronger ACLR groups, including the gait stance regions with statistically significant differences, the peak differences observed in those regions, and the corresponding average Cohen’s *d* effect sizes for the significant intervals. For effect size thresholds, see Methods. “Stronger” and “weaker” labels reflect relative quadriceps strength trajectory profiles within the ACLR cohort and do not indicate recovery to normative or clinically sufficient strength levels. Dashes indicate no statistically significant between-group differences detected for that comparison at the given timepoint (*P* ≥ 0.05). ACLR, anterior cruciate ligament reconstruction; KFA, knee flexion angle.

#### Knee Extension Moment

**Figure 4.**
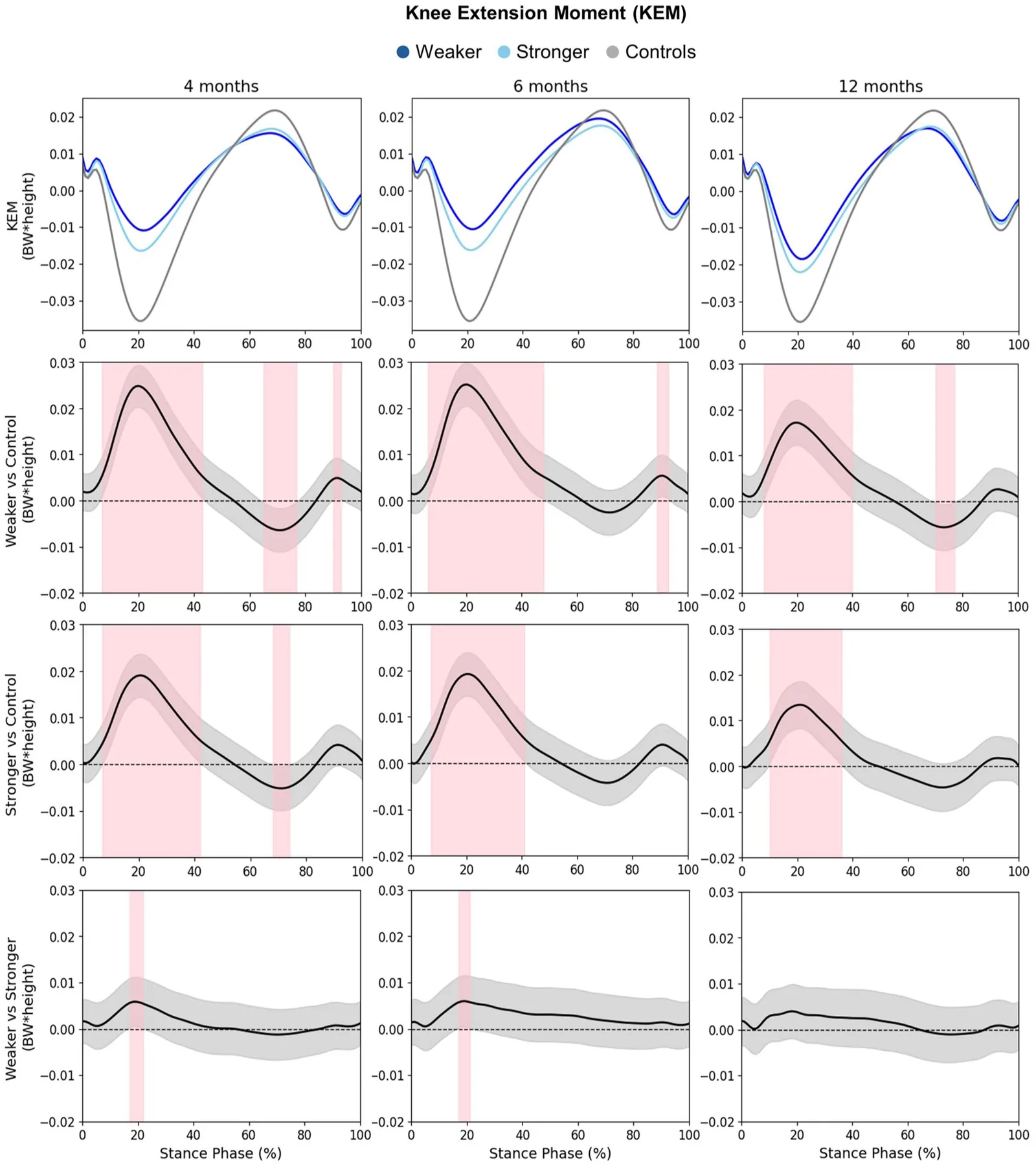
Average KEM waveforms and corresponding mean differences with 95% confidence intervals (shaded in gray) are presented across the gait stance phase for the weaker ACLR group, stronger ACLR group, and uninjured control group at the 4-, 6-, and 12-month timepoints after ACLR. The first row displays mean KEM waveforms for each group; the second row shows differences between the weaker ACLR group and controls; the third row compares the stronger ACLR group with controls; and the fourth row compares the weaker and stronger ACLR groups. The first, second, and third columns correspond to the 4-, 6-, and 12-month assessments, respectively. Statistically significant group differences, defined as intervals where the 95% CIs of the mean differences do not include zero, are highlighted in pink. ACLR, anterior cruciate ligament reconstruction; BW, body weight; KEM, knee extension moment.

The weaker group exhibited lower KEM during early stance at 4 (17% to 22%) and 6 (17% to 21%) months compared with the stronger ACLR group (Figure 4 and Table 5). Both the weaker and stronger ACLR groups showed lower KEM during the first half of stance compared with healthy controls at 4, 6, and 12 months post-ACLR. Both groups also showed lesser knee flexion moment during mid- to late-stance relative to controls. Notably, only the weaker ACLR group exhibited lesser KEM during late stance at 4 months (90% to 93%) and 6 months (89% to 93%) compared with healthy controls.

**Table 5.** Results of waveform analyses of KEM at 4-, 6-, and 12-month timepoints after ACLR

Comparison	Timepoint	Areas of difference, %	Maximum difference, BW × height	Cohen’s *d* effect size
(1) Weaker – controls	4 months	7-43	0.029	1.98
		65-77	–0.011	–0.75
		90-93	0.001	1.04
	6 months	6-48	0.025	0.28
		89-93	0.005	0.25
	12 months	8-40	0.017	1.33
		70-77	–0.005	–0.66
(2) Stronger – controls	4 months	7-42	0.019	1.47
		68-74	–0.005	-0.60
	6 months	7-41	0.019	1.50
	12 months	10-36	0.013	0.99
(3) Weaker – stronger	4 months	17-22	0.006	0.53
	6 months	17-21	0.006	0.51
	12 months	-	-	-

Comparisons were made between (1) the weaker ACLR group and healthy controls, (2) the stronger ACLR group and healthy controls, and (3) the weaker and stronger ACLR groups, including the gait stance regions with statistically significant differences, the peak differences observed in those regions, and the corresponding average Cohen’s *d* effect sizes for the significant intervals. For effect size thresholds, see Methods. “Stronger” and “weaker” labels reflect relative quadriceps strength trajectory profiles within the ACLR cohort and do not indicate recovery to normative or clinically sufficient strength levels. Dashes indicate no statistically significant between-group differences for that comparison at the given timepoint (*P* ≥ 0.05). ACLR, anterior cruciate ligament reconstruction; BW, body weight; KEM, knee extension moment.

#### Knee Adduction Moment

**Figure 5.**
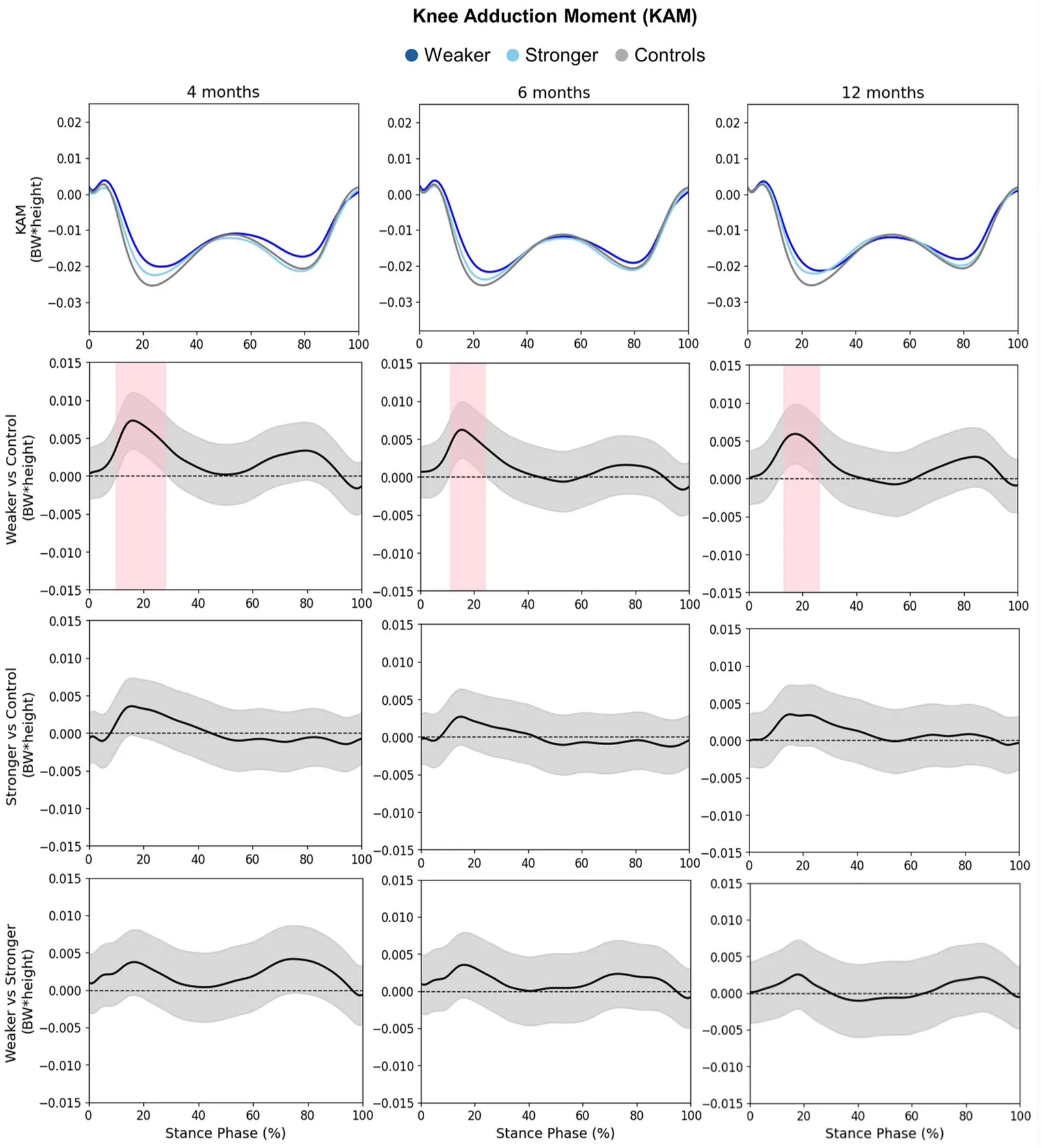
Average KAM waveforms and corresponding mean differences with 95% confidence intervals (shaded in gray) are presented across the gait stance phase for the weaker ACLR group, stronger ACLR group, and uninjured control group at the 4-, 6-, and 12-month timepoints after ACLR. The first row displays mean KAM waveforms for each group; the second row shows differences between the weaker ACLR group and controls; the third row compares the stronger ACLR group with controls; and the fourth row compares the weaker and stronger ACLR groups. The first, second, and third columns correspond to the 4-, 6-, and 12-month assessments, respectively. Statistically significant group differences, defined as intervals where the 95% CIs of the mean differences do not include zero, are highlighted in pink. ACLR, anterior cruciate ligament reconstruction; BW, body weight; KAM, knee adduction moment.

The weaker ACLR group showed lower KAM during early stance at 4 (10% to 28%), 6 (11% to 24%), and 12 (12% to 25%) months compared with healthy controls (Figure 5 and Table 6). No statistically significant differences were found at any other point in the stance phase between the weaker group and controls, nor were any differences observed between the stronger group and controls, or between the weaker and stronger groups.

**Table 6.** Results of waveform analyses of KAM at 4-, 6-, and 12-month timepoints after ACLR

Comparison	Timepoint	Areas of difference, %	Maximum difference, BW × height	Cohen’s d effect size
(1) Weaker – controls	4 months	10-28	0.007	1.08
	6 months	11-24	0.006	0.93
	12 months	12-25	0.006	0.84
(2) Stronger – controls	4 months	-	-	-
	6 months	-	-	-
	12 months	-	-	-
(3) Weaker – stronger	4 months	-	-	-
	6 months	-	-	-
	12 months	-	-	-

Comparisons were made between (1) the weaker ACLR group and healthy controls, (2) the stronger ACLR group and healthy controls, and (3) the weaker and stronger ACLR groups, including the gait stance regions with statistically significant differences, the peak differences observed in those regions, and the corresponding average Cohen’s *d* effect sizes for the significant intervals. For effect size thresholds, see Methods. “Stronger” and “weaker” labels reflect relative quadriceps strength trajectory profiles within the ACLR cohort and do not indicate recovery to normative or clinically sufficient strength levels. Dashes indicate no statistically significant between-group differences for that comparison at the given timepoint (*P* ≥ 0.05). ACLR, anterior cruciate ligament reconstruction; BW, body weight; KAM, knee adduction moment.

#### Gait Biomechanical Outputs With Gait Speed Adjustment

The results of the waveform analysis adjusted for gait speed are presented in the Supplemental Content document (Figures S1-S4 and Tables S1-S4, available in the online version of this article). Overall, adjusting for gait speed produced similar trends in the biomechanical outcomes analyzed in this study; most statistically significant differences in biomechanical waveforms persisted across groups. For vGRF, significant differences between the weaker ACLR group and controls at 4 months remained in early and late stance. For KFA, all between-group differences involving the weaker group versus controls and stronger group versus controls remained across early- to mid- to late-stance phases. Differences between the weaker and stronger ACLR groups also persisted during early stance at 6 months and 12 months. For KEM, significant differences between ACLR groups and controls remained during early stance across all timepoints. Additional differences between the weaker group and controls at mid- to late-stance (4 and 12 months) and between the stronger group and controls at mid- to late-stance (4 months) also persisted. Finally, for KAM, significant differences between the weaker group and controls at 4 months remained postadjustment.

## Discussion

Consistent with our hypothesis, and after accounting for uninjured limb strength, we identified a trajectory that demonstrated greater strength and a trajectory exhibiting more persistent weakness in the first 12 months post-ACLR (Figure 1). Importantly, these trajectory labels reflect relative differences in strength within the cohort rather than attainment of clinically sufficient quadriceps strength levels. Although both ACLR groups exhibited altered gait biomechanics at habitual walking speed, marked by less dynamic waveforms in vGRF, KFA, KEM, and KAM, the gait patterns of the stronger group more closely resembled those of healthy controls relative to the waveforms demonstrated by the weaker group. These findings suggest that while people with better quadriceps strength recovery did not fully restore normal gait patterns, strength recovery was associated with better gait biomechanics during the first year post-ACLR. Nevertheless, although restoring strength should remain a key focus of rehabilitation, incorporating additional interventions within the first year post-ACLR to specifically address gait biomechanics may be necessary to achieve full restoration of normal walking mechanics after ACL injury.

Previous cross-sectional studies have shown that achieving a quadriceps strength threshold of ≥3.0 Nm·kg^–1^ in MVIC is associated with better knee-related function and patient-reported outcomes after ACLR.^27^ A previous study assessing walking biomechanics of patients ≥12 months post-ACLR found that 21% of participants exceeded the 3.0 Nm·kg^–1^ threshold and exhibited gait profiles that differed from those who remained below it. Particularly, the altered walking biomechanics observed in the lower-strength group could be associated with KOA development.^26^ In the current study, average strength values for both trajectory groups (i.e., stronger and weaker) remained below the 3.0 Nm·kg^–1^ threshold, and neither returned to preoperative levels on average. Notably, although only 3 participants (5.1%) exceeded this threshold, between-group differences were still observed, consistent with findings from the previously reported cohort, in which a greater proportion of participants exceeded the strength threshold.^26^ Together, these findings suggest that the first 12 months after ACLR, a period during which most people have completed formal rehabilitation and returned to physical activity, might not be sufficient to recover adequate quadriceps strength,^18^ and that this persistent weakness may be associated with the aberrant walking biomechanics observed in both ACLR strength groups within the first year post-ACLR.^9^

Gait biomechanics in the stronger group were generally more similar to healthy controls than the weaker ACLR group was to healthy controls. For example, the weaker group demonstrated less dynamic vGRF profiles, characterized by greater vGRF during midstance at all timepoints and lower vGRF during late stance at 4 months, relative to controls (Figure 2). However, no statistically significant vGRF differences were observed between the stronger and weaker ACLR groups, suggesting that vGRF may not be influenced strongly by the observed changes in strength. The weaker group exhibited lesser knee flexion during the first half of stance compared with the stronger group at 6 months and 12 months (Figure 3), a pattern that may reduce the tibiofemoral contact area during the loading response, leading to more localized joint tissue loading.^1^ Furthermore, the stronger group demonstrated greater internal KEM at 4 and 6 months compared with the weaker group during the first half of the stance (Figure 4). Similarly, only the weaker group exhibited KAM differences compared with controls, with lower KAM during early stance at all timepoints (Figure 5), a pattern consistent with previous work linking reduced knee joint loading early after ACLR with subsequent radiographic KOA.^34^ No significant KAM differences were detected between the stronger and weaker ACLR groups, suggesting that observed improvements in strength do not result in substantial changes in KAM during gait. The distinct patterns of knee kinetics across ACLR strength groups during the first half of the stance, when the limb undergoes the greatest loading, may be linked to differences in loading in the ACLR trajectory groups; however, these differences were less pronounced in the stronger group than in the weaker group when compared with controls.

Gait speed also differed between the ACLR strength groups and healthy controls, as both the stronger and weaker ACLR groups were slower at all 3 timepoints (4, 6, and 12 months) compared with healthy controls (Table 2). When adjusting for gait speed, fewer regions of the stance phase showed statistically significant differences in biomechanical outcomes; however, most between-group differences persisted. This suggests that, while gait speed partially contributes to the observed gait differences, it does not fully explain them. This supports previous research indicating that simply increasing walking speed is insufficient to normalize gait waveforms in patients post-ACLR.^8^ Although not statistically significant, lateral and medial meniscal injuries were more frequent in the weaker group (77.4% and 29.0%, respectively) compared with the stronger group (57.1% and 17.9%, respectively). Previous studies have shown negative associations between meniscal repair and muscle strength.^13^ The Tegner activity level scale also yielded lower scores in the weaker group at 6 and 12 months (5.28 ± 1.81 and 7.14 ± 2.12, respectively) compared with the stronger group (6.04 ± 2.13 and 7.81 ± 2.33, respectively), aligning with previous research linking higher quadriceps strength to greater physical activity levels.^31^ Together, these trends suggest that meniscal injury and reduced activity engagement may contribute to weaker strength recovery trajectories after ACLR.

When considering interlimb symmetry, the stronger ACLR group demonstrated approximately 20% higher symmetry across time compared with the weaker ACLR group. Notably, despite achieving a mean LSI of 91.1 ± 11.7% at 12 months post-ACLR, people in the stronger group continued to exhibit gait biomechanics that did not fully resemble those of uninjured controls. The weaker trajectory group exhibited more pronounced gait abnormalities, indicating that persistent quadriceps weakness may help identify people at greater risk of aberrant loading. However, because only a few participants achieved higher strength thresholds, potentially due to arthrogenic muscle inhibition limiting voluntary activation,^28^ strength recovery alone may be insufficient to fully restore normal gait biomechanics.^2,26^ These findings underscore the need for complementary, targeted strategies to address residual biomechanical deficits that remain despite adequate strength recovery within the first year post-ACLR. Motor learning-based gait therapies, such as real-time gait biofeedback,^5,14^ represent one potential avenue to complement traditional strength training by directly retraining aberrant loading patterns.^5,14,22^ This approach has been shown to increase dynamic loading, resulting in more dynamic tibiofemoral contact forces during walking and a positive acute biological response to a bout of loading,^3,23^ thereby serving as a complementary strategy to strength rehabilitation and potentially mitigating long-term KOA risk.

While this is the first study comparing walking gait profiles between ACLR strength trajectory groups collected over the first 12 months post-ACLR, some limitations should be acknowledged. First, we did not assess gait biomechanics before ACL injury, so we are unable to determine whether there were intrinsic differences between ACLR strength group participants and controls before the injury. Moreover, as assessments were limited to 12 months by design, the persistence of aberrant biomechanics beyond this period remains unknown. Because the majority of participants (93.2%) received a bone-patellar tendon-bone autograft, our findings may not be generalizable to patients receiving other graft types. Rehabilitation protocols were not standardized; however, our cohort may be more generalizable to current rehabilitative practices. Finally, although meniscal and chondral injuries were reported, injury severity was not included in the analysis, as it did not differ significantly between strength trajectory groups. In our cohort, 24 participants underwent meniscectomy, whereas 29 underwent meniscal repairs. We did not stratify analyses by meniscal treatment; however, because repairs may involve prolonged weightbearing restrictions, future studies with sufficient statistical power should examine how treatment type influences strength recovery and gait biomechanics.

Overall, this study identified 2 distinct quadriceps strength recovery trajectories, stronger and weaker groups, after ACLR. While the stronger group exhibited more dynamic patterns than the weaker group, it did not fully normalize walking biomechanics to those of healthy controls. These findings suggest that, in addition to strength training, rehabilitation strategies that target gait retraining may be needed to address aberrant gait biomechanics to mitigate risk factors associated with KOA development.

## Supplemental Material

sj-docx-1-sph-10.1177_19417381261479680 – Supplemental material for Effects of Quadriceps Strength Recovery in the First Year After Anterior Cruciate Ligament Reconstruction on Gait Biomechanics Supplemental material, sj-docx-1-sph-10.1177_19417381261479680 for Effects of Quadriceps Strength Recovery in the First Year After Anterior Cruciate Ligament Reconstruction on Gait Biomechanics by Sergio A. Lemus, Christin Büttner, Liubov Arbeeva, Caroline Lisee, Natália Favoreto, R. Alexander Creighton, Ganesh M. Kamath, Jeffrey T. Spang, Christopher D. Ingersoll, Samantha Tayne, Joe M. Hart, J. Troy Blackburn and Brian Pietrosimone in Sports Health
