# Supplementary material for: Effects of Quadriceps Strength Recovery in the First Year After Anterior Cruciate Ligament Reconstruction on Gait Biomechanics
Source: Sports Health. 2026 Sep 11:19417381261479680. Online ahead of print. doi: 10.1177/19417381261479680 (PMC13570954; doi:10.1177/19417381261479680)
Supplement: Supplementary file 1 [file sj-docx-1-sph-10.1177_19417381261479680.docx]

**Supplemental Content**

**Supplemental Figures**

**Supplemental Figure 1** – Average vertical ground reaction force (vGRF) waveforms adjusted for gait speed, along with corresponding mean differences and 95% confidence intervals (shaded in gray), are presented across the gait stance phase for the weaker anterior cruciate ligament reconstruction (ACLR) group, stronger ACLR group, and uninjured control group at the 4-, 6-, and 12-month timepoints following ACLR. The first row displays the mean vGRF waveforms for each group; the second row shows differences between the weaker ACLR group and controls; the third row compares the stronger ACLR group to controls; and the fourth row compares the weaker and stronger ACLR groups. The first, second, and third columns correspond to the 4-, 6-, and 12-month assessments, respectively. Statistically significant group differences—defined as regions where the 95% confidence intervals of the mean differences do not include zero—are highlighted in pink.

**Vertical Ground Reaction Force (vGRF)**

● Weaker	● Stronger	● Controls

**Supplemental Figure 2** – Average knee flexion angle (KFA) waveforms adjusted for gait speed, along with corresponding mean differences and 95% confidence intervals (shaded in gray), are presented across the gait stance phase for the weaker anterior cruciate ligament reconstruction (ACLR) group, stronger ACLR group, and uninjured control group at the 4-, 6-, and 12-month timepoints following ACLR. The first row displays the mean KFA waveforms for each group; the second row shows differences between the weaker ACLR group and controls; the third row compares the stronger ACLR group to controls; and the fourth row compares the weaker and stronger ACLR groups. The first, second, and third columns correspond to the 4-, 6-, and 12-month assessments, respectively. Statistically significant group differences—defined as regions where the 95% confidence intervals of the mean differences do not include zero—are highlighted in pink.

**Knee Flexion Angle (KFA)**

● Weaker	● Stronger	● Controls

**Supplemental Figure 3** – Average knee extension moment (KEM) waveforms adjusted for gait speed, along with corresponding mean differences and 95% confidence intervals (shaded in gray), are presented across the gait stance phase for the weaker anterior cruciate ligament reconstruction (ACLR) group, stronger ACLR group, and uninjured control group at the 4-, 6-, and 12-month timepoints following ACLR. The first row displays the mean KEM waveforms for each group; the second row shows differences between the weaker ACLR group and controls; the third row compares the stronger ACLR group to controls; and the fourth row compares the weaker and stronger ACLR groups. The first, second, and third columns correspond to the 4-, 6-, and 12-month assessments, respectively. Statistically significant group differences—defined as regions where the 95% confidence intervals of the mean differences do not include zero—are highlighted in pink.

**Knee Extension Moment (KEM)**

● Weaker	● Stronger	● Controls

**Supplemental Figure 4** – Average knee adduction moment (KAM) waveforms adjusted for gait speed, along with corresponding mean differences and 95% confidence intervals (shaded in gray), are presented across the gait stance phase for the weaker anterior cruciate ligament reconstruction (ACLR) group, stronger ACLR group, and uninjured control group at the 4-, 6-, and 12-month timepoints following ACLR. The first row displays the mean KAM waveforms for each group; the second row shows differences between the weaker ACLR group and controls; the third row compares the stronger ACLR group to controls; and the fourth row compares the weaker and stronger ACLR groups. The first, second, and third columns correspond to the 4-, 6-, and 12-month assessments, respectively. Statistically significant group differences—defined as regions where the 95% confidence intervals of the mean differences do not include zero—are highlighted in pink.

**Knee Adduction Moment (KAM)**

● Weaker	● Stronger	● Controls

**Supplemental Tables**

**Supplemental Table 1. Results from the waveform analyses of vertical ground reaction force (vGRF), adjusted for gait speed, are presented at 4-, 6-, and 12-month timepoints following anterior cruciate ligament reconstruction (ACLR).** Comparisons were conducted between (a) the weaker ACLR group and healthy controls, (b) the stronger ACLR group and healthy controls, and (c) the weaker and stronger ACLR groups. The results include gait stance regions with statistically significant differences, peak differences observed within those regions, and the corresponding average Cohen’s *d* effect sizes for the significant intervals. Effect sizes were interpreted using the following thresholds: small (Cohen’s *d* ≤ 0.2), medium (0.2 < Cohen’s *d* ≤ 0.5), and large (Cohen’s *d* ≥ 0.8). “Stronger” and “weaker” labels reflect relative quadriceps strength trajectory profiles within the ACLR cohort and do not indicate recovery to normative or clinically sufficient strength levels. Dashes (–) indicate that no statistically significant between-group differences were detected for that comparison at the given timepoint (p ≥ 0.05).

| **Comparison** | **Timepoint** | **Areas of difference (%)** | **Maximum difference (bodyweight)** | **Cohen’s *d* effect size** |
| --- | --- | --- | --- | --- |
| Weaker-Controls | 4 months | 14-23 | -0.082 | -1.54 |
|  |  | 79-81 | -0.063 | -1.43 |
|  | 6 months | – | – | – |
|  | 12 months | – | – | – |
| Stronger-Controls | 4 months | – | – | – |
|  | 6 months | – | – | – |
|  | 12 months | – | – | – |
| Weaker-Stronger | 4 months | – | – | – |
|  | 6 months | – | – | – |
|  | 12 months | – | – | – |

**Supplemental Table 2. Results from the waveform analyses of the knee flexion angle (KFA), adjusted for gait speed, are presented at 4-, 6-, and 12-month timepoints following anterior cruciate ligament reconstruction (ACLR).** Comparisons were conducted between (a) the weaker ACLR group and healthy controls, (b) the stronger ACLR group and healthy controls, and (c) the weaker and stronger ACLR groups. The results include gait stance regions with statistically significant differences, peak differences observed within those regions, and the corresponding average Cohen’s d effect sizes for the significant intervals. Effect sizes were interpreted using the following thresholds: small (Cohen’s *d* ≤ 0.2), medium (0.2 < Cohen’s *d* ≤ 0.5), and large (Cohen’s *d* ≥ 0.8). “Stronger” and “weaker” labels reflect relative quadriceps strength trajectory profiles within the ACLR cohort and do not indicate recovery to normative or clinically sufficient strength levels. Dashes (–) indicate that no statistically significant between-group differences were detected for that comparison at the given timepoint (p ≥ 0.05).

| **Comparison** | **Timepoint** | **Areas of difference (%)** | **Maximum difference (°)** | **Cohen’s *d* effect size** |
| --- | --- | --- | --- | --- |
| Weaker-Controls | 4 months | 3-39 | -4.33 | -0.95 |
|  |  | 52-84 | 3.66 | 0.77 |
|  |  | 100 | -1.35 | -0.43 |
|  | 6 months | 0-46 | -5.70 | -1.12 |
|  |  | 65-74 | 1.66 | 0.48 |
|  |  | 98-100 | -1.78 | -0.43 |
|  | 12 months | 0-43 | -4.11 | -0.92 |
|  |  | 63-81 | 1.95 | 0.52 |
| Stronger-Controls | 4 months | 5-38 | -3.35 | -0.79 |
|  |  | 51-88 | 3.96 | 0.87 |
|  | 6 months | 0-40 | -4.01 | -0.78 |
|  |  | 55-84 | 2.95 | 0.73 |
|  |  | 100 | -1.28 | -0.32 |
|  | 12 months | 6-35 | -2.30 | -0.53 |
|  |  | 55-96 | 3.04 | 0.69 |
| Weaker-Stronger | 4 months | – | – | – |
|  | 6 months | 13-17 | -1.70 | -0.34 |
|  | 12 months | 10-18 | -2.05 | -0.45 |

**Supplemental Table 3. Results from the waveform analyses of the knee extension moment (KEM), adjusted for gait speed, are presented at 4-, 6-, and 12-month timepoints following anterior cruciate ligament reconstruction (ACLR).** Comparisons were conducted between (a) the weaker ACLR group and healthy controls, (b) the stronger ACLR group and healthy controls, and (c) the weaker and stronger ACLR groups. The results include gait stance regions with statistically significant differences, peak differences observed within those regions, and the corresponding average Cohen’s *d* effect sizes for the significant intervals. Effect sizes were interpreted using the following thresholds: small (Cohen’s *d* ≤ 0.2), medium (0.2 < Cohen’s *d* ≤ 0.5), and large (Cohen’s *d* ≥ 0.8). “Stronger” and “weaker” labels reflect relative quadriceps strength trajectory profiles within the ACLR cohort and do not indicate recovery to normative or clinically sufficient strength levels. Dashes (–) indicate that no statistically significant between-group differences were detected for that comparison at the given timepoint (p ≥ 0.05).

| **Comparison** | **Timepoint** | **Areas of difference (%)** | **Maximum difference (bodyweight *height)** | **Cohen’s *d* effect size** |
| --- | --- | --- | --- | --- |
| Weaker-Controls | 4 months | 6-41 | 0.019 | 2.02 |
|  |  | 64-78 | -0.007 | -0.75 |
|  | 6 months | 6-47 | 0.020 | 1.99 |
|  | 12 months | 8-40 | 0.018 | 1.40 |
|  |  | 73-76 | -0.005 | -0.65 |
| Stronger-Controls | 4 months | 7-40 | 0.015 | 1.51 |
|  |  | 67-76 | -0.006 | -0.60 |
|  | 6 months | 6-40 | 0.016 | 1.53 |
|  | 12 months | 10-34 | 0.010 | 1.01 |
| Weaker-Stronger | 4 months | – | – | – |
|  | 6 months | – | – | – |
|  | 12 months | – | – | – |

**Supplemental Table 4. Results from the waveform analyses of the knee adduction moment (KAM), adjusted for gait speed, are presented at 4-, 6-, and 12-month timepoints following anterior cruciate ligament reconstruction (ACLR).** Comparisons were conducted between (a) the weaker ACLR group and healthy controls, (b) the stronger ACLR group and healthy controls, and (c) the weaker and stronger ACLR groups. The results include gait stance regions with statistically significant differences, peak differences observed within those regions, and the corresponding average Cohen’s *d* effect sizes for the significant intervals. Effect sizes were interpreted using the following thresholds: small (Cohen’s *d* ≤ 0.2), medium (0.2 < Cohen’s *d* ≤ 0.5), and large (Cohen’s *d* ≥ 0.8). “Stronger” and “weaker” labels reflect relative quadriceps strength trajectory profiles within the ACLR cohort and do not indicate recovery to normative or clinically sufficient strength levels. Dashes (–) indicate that no statistically significant between-group differences were detected for that comparison at the given timepoint (p ≥ 0.05).

| **Comparison** | **Timepoint** | **Areas of difference (%)** | **Maximum difference (bodyweight *height)** | **Cohen’s d effect size** |
| --- | --- | --- | --- | --- |
| Weaker-Controls | 4 months | 12-18 | 0.005 | 1.09 |
|  | 6 months | – | – | – |
|  | 12 months | – | – | – |
| Stronger-Controls | 4 months | – | – | – |
|  | 6 months | – | – | – |
|  | 12 months | – | – | – |
| Weaker-Stronger | 4 months | – | – | – |
|  | 6 months | – | – | – |
|  | 12 months | – | – | – |
